# Polygenic Associations With Educational Attainment in East Versus West Germany: Differences Emerge After Reunification

**DOI:** 10.1177/09567976251350965

**Published:** 2025-07-09

**Authors:** Deniz Fraemke, Yayouk E. Willems, Aysu Okbay, Ulman Lindenberger, Sabine Zinn, Gert Wagner, David Richter, Kathryn P. Harden, Elliot M. Tucker-Drob, Ralph Hertwig, Philipp Koellinger, Laurel Raffington

**Affiliations:** 1 Max Planck Research Group Biosocial – Biology, Social Disparities, and Development, Max Planck Institute for Human Development, Berlin, Germany; 2 School of Business and Economics, Tinbergen Institute, Amsterdam; 3 Amsterdam Neuroscience, Complex Trait Genetics, Vrije Universiteit Amsterdam; 4 Department of Economics, School of Business and Economics, Vrije Universiteit Amsterdam; 5 Max Planck Institute for Human-Development, Berlin, Germany; 6 German Socio-Economic Panel Study Department; 7 Department of Social Sciences, Humboldt University, Berlin; 8 Department of Education and Psychology, Freie Universität zu Berlin; 9 SHARE Berlin; 10 Department of Psychology, The University of Texas, US; 11 Population Research Center, The University of Texas, US

**Keywords:** education, genetics, gene–environment interaction, German reunification, polygenic index

## Abstract

Using a DNA-based polygenic index, we explored geographical and historical differences in polygenic associations with educational attainment in East and West Germany around the time of reunification. This index was derived from a prior genome-wide association study on educational attainment in democratic countries. In 1,930 individuals aged 25 to 85 years from the SOEP-G[ene] cohort, the magnitude of polygenic associations with educational attainment did not differ between East and West Germany before reunification but increased in East Germany thereafter. This gene–environment interaction remained robust when we probed for variance dispersion. A control analysis using a polygenic index of height suggests that this interaction is unlikely to reflect a general trend toward greater genetic associations in East Germany after reunification. The observed amplification of education-genetic associations aligns with theories suggesting heightened genetic influences on educational attainment during periods of greater social and educational opportunity. We emphasize the need for replication in larger German genetic data sets.

## Introduction

Educational performance has long been known to be heritable, meaning that more genetically similar relatives resemble each other more in their educational outcomes. Recently, large-scale molecular studies have identified specific genetic variants that are correlated with educational attainment, defined as years of formal schooling. However, genes are not deterministic of social and behavioral outcomes like educational performance and instead are related probabilistically to outcomes via mechanisms that depend on environmental inputs. Consequently, genetic associations are expected to vary across historical time and social context. Here, we leverage data that span a profound social transition—the German reunification—in order to test how genetic associations with educational performance differ across time and place.

After World War II, Germany was divided into two separate states: East Germany became a member of the Soviet-controlled Warsaw Pact, and West Germany became a member of the North Atlantic Treaty Organization, allied with Western democracies. The state-socialist East German regime enforced large-scale institutional reforms aimed at transforming the social, economic, and educational systems to foster opportunities for the working class and reduce intergenerational educational inequality. Accordingly, the educational systems of East and West Germany differed significantly in terms of intergenerational inequality and ideological influence (Anweiler, 1990; Fischer, 1992; von Below, 2002). The East German system prioritized children of industrial and agricultural workers, introduced a comprehensive school until the 10th grade, and aimed to provide more academic support for low-achieving students (Anweiler, 1990; von Below, 2017). Furthermore, ideological discrimination was integrated into admission requirements for higher education (Anweiler, 1989; Fuchs-Schündeln & Masella, 2016). Beyond academic performance, students were expected to exhibit a “socialist personality” and align politically with the ruling party. This ideological discrimination has been linked to reduced polygenic associations with educational attainment during state socialism (Rimfeld et al., 2018).

In contrast, the West German educational system was (and largely remains) characterized by ideological pluralism and early school tracking. This early tracking process combines performance-based assessments with input from parents and teachers to place children into hierarchically structured school tracks around the age of 10 years (Anweiler, 1990). In quasiexperimental designs, tracking has been shown to perpetuate intergenerational inequality by amplifying parent-offspring educational similarity (Lange & von Werder, 2017). Thus, school tracking may partially explain the reduced genetic influence on educational attainment in German twin studies compared with studies in more liberal educational systems, such as Norway or Sweden (Baier et al., 2022; Tucker-Drob & Bates, 2016); (See Box 1 in the Supplemental Material available online for further information on East–West differences in educational systems). With the collapse of European state socialism in 1989 and 1990, East Germany’s educational ideology was swiftly replaced by West Germany’s pluralist and meritocratic ideology, which was oriented toward free-market productivity (Littler, 2017; Rohde, 2023; Solga, 2005).

Here we explore geographical and historical differences in genetic associations with educational attainment in East and West Germany around the time of reunification. Our study includes 1,930 individuals aged 25 to 85 years from the German SOEP-G[ene] cohort, of which 460 lived in East Germany and 1,470 lived in West Germany between 1934 and 2020. We utilize a DNA-based summary measure of genetic influence known as the *polygenic index of educational attainment* (PGI-Education). This polygenic index aggregates weighted allele counts that were previously associated with educational attainment in a genome-wide association study (GWAS) of 3,037,499 individuals, of which 98.6% completed their education in free-market democracies (EA4; Okbay et al., 2022). Consequently, the weights used in calculating PGI-Education are reflective of dynamics in Western democratic countries, which may not be applicable to other contexts, such as those in European states under the Soviet Union (Rimfeld et al., 2018; Ujma et al., 2022). We test for such differential associations with PGI-education in East and West Germany around the time of reunification.

## Research Transparency Statement

### General disclosures

**Conflicts of interest:** All authors declare no conflicts of interest. **Funding:** During the work on this article, D. Fraemke was a predoctoral fellow of the International Max Planck Research School on the Life Course (LIFE, www.imprs-life.mpg.de; participating institutions: Max Planck Institute for Human Development, Freie Universität Berlin, Humboldt-Universität zu Berlin, University of Michigan, University of Virginia, University of Zurich). Y. E. Willems has received funding from the European Union’s Horizon Europe research and innovation program under the Marie Skłodowska-Curie grant agreement (No. 101150809 – EpiSoDi). D. Fraemke, Y. E. Willems, U. Lindenberger, R. Hertwig, G. Wagner, and L. Raffington received funding from the Max Planck Society. K. P. Harden was supported by NIH Grants No. R01HD083613 and No. R01HD092548. K. P. Harden and E. M. Tucker-Drob are Faculty Research Associates of the Population Research Center (PRC), which is supported by National Institutes of Health (NIH) Grant No. P2CHD042849. E. M. Tucker-Drob was supported by NIH grants (Nos. RF1AG073593, R01MH120219, and R01HD092548). E. M. Tucker-Drob is Faculty Research Associate of the Center for Aging and Populations Sciences (CAPS), which is supported by NIH Grant No. P30AG066614. P. Koellinger received funding from the University of Amsterdam. U. Lindenberger, David Richter, R. Hertwig, and L. Raffington are faculty members at LIFE. K. P. Harden, E. M. Tucker-Drob, and L. Raffington were supported by fellowships from the Jacobs Foundation. L. Raffington is supported by the European Union, Project No. 101073237 – ESSGN. Funders had no role in the design and conduct of the study; collection, management, analysis, and interpretation of the data; preparation, review, or approval of the manuscript; and decision to submit the manuscript for publication. **Artificial intelligence:** No artificial-intelligence-assisted technologies were used in this research or the creation of this article. **Ethics:** The data collection for this study has received ethical approval by the Vrije Universiteit Amsterdam, School of Business and Economics (Application No. 20181018.1.pkr730) and by the Max Planck Society (Application No. 2019_16).

### Study disclosures

**Preregistration:** No aspect of this study was preregistered. **Materials:** The SOEP-IS is a longitudinal panel study initiated in 1998 that collects data annually. All questionnaires and summary statistics of each item used in this study are provided in the data documentation of the SOEP-IS (https://paneldata.org/soep-is/). **Data:** All data used in this study are fully accessible to eligible researchers upon application to the management of the DIW Berlin. For more details see https://osf.io/baehf. **Analysis scripts:** All analysis scripts are publicly available (https://osf.io/8skmg/). **Computational reproducibility:** The reproducibility of this manuscript was not verified by the journal’s STAR team because it was not possible to access the data within a reasonable time frame. The original analyses were run by D. Fraemke and then independently reproduced by Y. E. Willems.

## Method

### Participants

The Socio-Economic Panel (SOEP) is a population-based, multigenerational household survey study (Goebel et al., 2019). SOEP participants from infant age onward (*N* = 6,576) were randomly selected and invited to participate in buccal DNA genotyping as part of the SOEP-Gene subsample (SOEP-G; Koellinger et al., 2023). In total, genetic data are available for 2,262 adults (*M*_age_ = 56.13 years, *SD*_age_ = 18.72 years, 54% female), with 98% of participants showing high genetic similarity to European reference groups (see Koellinger et al., 2023). Polygenic analyses were restricted to participants with high genetic similarity to European reference groups in order to maximize similarity to the participants in the EA4 GWAS discovery sample, the results of which were used to calculate PGI-Education. This restriction lowers the risk of confounding from population stratification and avoids established problems in the portability of polygenic indices across genetic ancestry groups (Martin et al., 2017; Price et al., 2006). See the Supplemental Methods section in the Supplemental Material for information on DNA preprocessing.

Present analyses included 1,930 participants, 25 to 85 years old, from the German SOEP-G cohort, of which 460 lived in East Germany and 1,470 in West Germany between the years 1949 and 2020. We differentiate between individuals who underwent their formative school years before and after German reunification by applying an age cutoff of 15 years in 1990 (cf. Rimfeld et al., 2018), resulting in birth cohorts up to 1975. Among East Germans, this cutoff resulted in 353 individuals who turned 15 before the reunification and 107 after. Among West Germans, 1,141 turned 15 before reunification and 329 after. Individuals born before 1934 were excluded (*n* = 86), because they turned 15 before Germany was officially divided in 1949.

### Measures

Table 1 reports variables of interest. To improve the interpretability of the parameters, we *z*-standardized the variables years of education, PGI-Education, and birth year across all individuals in SOEP-G. Individuals older than 25 years who had missing information on years of education (*n* = 9) or region (*n* = 95) were subsequently excluded from the analyses. We examined group differences between East and West Germany in all continuous variables by conducting *t* tests and χ² tests for categorical variables with an alpha level of .05 for each test.

**Table 1.** Main Variables of Interest

Construct	Description
Educational attainment	Educational attainment was assessed in number of years of education an individual obtained on a scale ranging from “compulsory school degree” (min = 7 years) to “university degree” (max = 18 years). Tertiary education in years was added to the years spent in secondary education (e.g., +1.5 years for vocational training or +5 years for a university degree; Goebel et al., 2019).
Region (East vs. West Germany)	Whether an individual lived in East or West Germany during or after reunification was assessed by two items in the SOEP data set. The individuals indicated whether they lived in East or West Germany before the fall of the Berlin Wall in 1989 (ppfad/loc1989). For individuals who were born after or had missing data on this variable, their household location was used (hbrutto/sampreg; Goebel et al., 2019).
PGI-Education	The polygenic index of educational attainment was computed on the basis of the most recent genome-wide association study of educational attainment in a sample of ~ 3 million individuals who are highly similar in genetic ancestry to European reference groups (Okbay et al., 2022). PGI-Education was computed with SBayesR using the default settings (Lloyd-Jones et al., 2019). We residualized PGI-Education for the top 20 genetic principal components of genetic ancestry and controlled for genotype batch using random effects (Price et al., 2006).
PGI-Height	The polygenic index of height was computed on the basis of a prior genome-wide association study of height in a sample of 448,198 individuals (Koellinger et al., 2023). We residualized PGI-Height for the top 20 genetic principal components of genetic ancestry and controlled for genotype batch using random effects (Price et al., 2006).
Birth year	Self-reported birthdate was transformed into a continuous birth-year variable.
Reunification (prereunification vs. postreunification)	We differentiate between individuals who underwent formative school years before and after German reunification by applying an age cutoff of 15 years in 1990 on the basis of participants’ self-reported birthday (cf. Rimfeld et al., 2018).

### Statistical procedure

We compared East–West differences in polygenic associations with educational attainment before and after the German reunification in 1990. We implemented stepwise multiple regressions estimated using the *stats* package in the R software (R Core Team, 2024). We specified a testwise alpha level of .05. Because we tested only one focal hypothesis, we did not implement familywise error corrections, which can substantially reduce statistical power.

We then further probed this hypothesis in a second set of analyses with an alternative specification of the same phenomenon. Specifically, we examined continuous linear differences in East–West polygenic associations with educational attainment across birth-year cohorts. Third, we conducted negative control analyses to probe whether effects were specific to the educational domain using a polygenic index of height (PGI-Height). In addition, we performed sensitivity analyses to probe the robustness of gene–environment interaction effects, which are primarily reported in the Supplemental Results in the Supplemental Material. These analyses included heteroscedasticity models to examine whether an interaction had occurred through dispersion in the variance of years of education (Domingue et al., 2022) and an analysis of potential differences in the means and distributions of PGI-Education between East and West Germany. No aspect of this study was preregistered.

#### Reunification analyses

In a first step, we examined whether the magnitude of the association between PGI-Education and years of education differed before and after German reunification. We regressed years of education on PGI-Education, reunification, and the interaction term PGI-Education × Reunification (Model 1). In a second step, we examined whether the magnitude of the association between PGI-Education and years of education differed between East and West Germany in the full sample. We regressed years of education on PGI-Education, region, and the interaction term PGI-Education × Region (Model 2). In a third step, we examined whether genetic associations with years of education after reunification differed between East and West Germany. We regressed years of education on PGI-Education, reunification, region, the two-way interaction terms PGI-Education × Reunification, PGI-Education × Region, and Reunification × Region as well as the focal three-way interaction PGI-Education × Reunification × Region (Model 3).

#### Birth-year analyses

Since German reunification denotes a single historical event, it can be considered a historically bound categorical version of birth year. Thus, we examined whether the gene–environment interaction with reunification replicated using a birth-year variable. We ran the same models as those listed in the reunification analyses, replacing the reunification (prereunification vs. postreunification) variable with the continuous variable birth year (Model 1b and Model 3b).

The educational reforms around reunification affected all students, with younger students in 1990 likely experiencing a more significant effect from the new system. Grouping of individuals on the basis of a 15-year cutoff does not adequately capture the gradual nature of this transition. To explore continuous differences in the association between PGI-Education and years of education across birth cohorts, we performed locally weighted regressions using the *np* package in R (Hayfield & Racine, 2008). This nonparametric method captures the nonlinear transitional nature across cohorts by assigning greater weight to individuals born nearer to the focal birth year through a Gaussian kernel function, enhancing the precision of local estimates. The bandwidth of the weighting kernel was determined using least-squares cross-validation, effectively balancing the smoothness and fit of the regression curve (Li & Racine, 2007).

#### Negative control analysis with PGI-Height

To assess whether findings were unique to the educational domain or indicated a more general pattern of varying genetic associations over time, we conducted negative control analyses using a polygenic index of height (PGI-Height). We replicated the models from the reunification analysis, substituting PGI-Education with PGI-Height and probing its association with both years of education and self-reported height.

#### Covariates

Gender differences in educational attainment have been reported across examined historical periods and regions and might confound estimates of gene–environment interaction (Hadjar & Berger, 2010; Herd et al., 2019). Therefore, we included self-reported gender as a covariate in all models, including the necessary Gender × Environment and Gender × Gene interaction terms (cf. Keller, 2014). For models that estimated interactions with time (reunification or birth year), we added the three-way interactions Gender × Gene × Environment, Gender × Gene × Time Period and Gender × Environment × Time (Models 3 and 3b).

#### Heteroscedasticity

Gene–environment interactions can emerge through multiple mechanisms, including a bias introduced when the variance of the dependent variable is moderated by either the environment or the genetic predictor (Westerman & Sofer, 2024). Domingue et al. (2022) developed a method to directly model the dispersion of the outcome and test whether a gene-environment interaction is specific to a measured predictor or represents a general pattern of variation in the outcome. We applied this method and estimated an environmental and a genetic heteroscedasticity model for each significant two-way interaction of interest. These models extend the general linear model with a coefficient (λ_E_ or λ_G_), estimating the interaction between the error term and the respective environmental or genetic predictor, which indexes heteroscedasticity. Subsequently, we derived the test statistics *ξ*_E_ and *ξ*_G_ from the corresponding models (see Supplemental Methods in the Supplemental Material). If a χ² test fails to reject *H*_0_ : ξ = 0, it is possible that the interaction is driven by dispersion in the outcome over the respective environmental or genetic predictor. When the test suggests rejection of *H*_0_ : ξ = 0, alternative forms of gene–environment interactions are implicated. For significant three-way interactions, we split the sample and tested whether it is possible that the underlying significant two-way interactions arose because of dispersion in years of education. Significant χ² tests of these two-way interactions indicate that the three-way interaction building on it is not occurring through heteroscedasticity. We set the significance level to .05 and conducted this analysis using the publicly available code (Domingue et al., 2022).

## Results

Descriptive statistics on East–West differences for all variables are reported in Table 2. There were no statistically significant mean differences in the dependent variable, years of education, between East and West Germans, *t*(828.1) = −1.03, *p* = .303 (see Fig. S1 in the Supplemental Material). Moreover, we did not observe significant group differences between East and West Germans on the variables PGI-Education, *t*(838.47) = 0.80, *p* = .422, birth year, *t*(789) = 0.87, *p* = .387, the proportion of females, χ²(1, *N* = 1,938) < 0.001, *p* = .999, or the proportion of genetic samples that did not pass strict quality-control exclusionary criteria, χ²(1, *N* = 1,777) = 0.31, *p* = .578.

**Table 2.** Descriptive Statistics of Variables in East and West Germans

Variable	West			East		
	*M*	*SD*	95% CI	*M*	*SD*	95% CI
Female (in %)	54.22			54.13		
Birth year	1961.3	16.26	[1934, 1994]	1960.6	15.75	[1934, 1994]
PGI-Education	0.01	0.98	[−3.9, 3.5]	−0.05	0.98	[−3, 3.3]
Years of education	12.52	2.76	[7, 18]	12.66	2.54	[7, 18]
Strict QC pass (in %)	91.50			92.39		

Note: PGI-Education was *z*-standardized across all individuals in SOEP-G that provided genetic data. None of the East–West differences were statistically significant at α = .05. QC = quality control of genetic data; CI = confidence interval.

### Reunification analyses

First, we examined whether the magnitude of the association between PGI-Education and educational attainment differed before and after German reunification. Across the whole sample, genetic associations with educational attainment were stronger post- compared to pre-reunification (see Model 1 in Table 3). This gene–environment interaction remained significant when applying environmental and genetic heteroscedasticity models (Table 4).

**Table 3.** Model Parameter Estimates Including Models With PGI-Education, German Reunification, and Region

Term	β	*SE*	*p*	95% CI
Model 1: PGI-Education × Reunification				
PGI-Education	0.28	0.03	< .001***	[0.22, 0.34]
Reunification (postreunification)	0.46	0.07	< .001***	[0.33, 0.60]
PGI-Education × Reunification (post)	0.15	0.05	.003**	[0.05, 0.26]
N = 2,025; N_pre_ = 1,561, N_post_ = 464.				
Model 2: PGI-Education × East vs. West Germany				
PGI-Education	0.33	0.03	< .001***	[0.27, 0.40]
Region (East Germany)	0.09	0.07	.217	[−0.05, 0.23]
PGI-Education × Region (East Germany)	0.04	0.05	.384	[−0.06, 0.15]
*N* = 1,930; *n*_east_ = 460, *n*_west_ = 1,470				
Model 3: PGI-Education × Reunification × East vs. West Germany				
PGI-Education	0.29	0.04	<.001***	[0.22, 0.36]
Region (East Germany)	0.10	0.08	.178	[−0.05, 0.26]
Reunification (Post)	0.46	0.08	< .001***	[0.30, 0.62]
PGI-Education × Region (East Germany)	−0.04	0.07	.551	[−0.19, 0.10]
Reunification (Post) × Region (East Germany)	0.15	0.17	.402	[−0.19, 0.48]
PGI-Education × Reunification (Post)	0.10	0.08	.212	[−0.06, 0.26]
PGI-Education × Reunification (Post) × Region (East Germany)	0.28	0.13	.025*	[0.04, 0.53]
*N* = 1,930; prereunification: *n*_west_ = 1,141; *n*_east_ = 353; postreunification: *n*_west_ = 329; *n*_east_ = 107.				

***p < .001. **p < .01. *p < .05.

**Table 4.** Heteroscedasticity Model Parameter Estimates

Term	τ_1_	π_0_	π_1_	λ_0_	λ_E_	λ_G_	ξ_E_	Pr(ξ_E_)	ξ_G_	Pr(ξ_G_)
Reunification analyses										
PGI-Education × Reunification	0.26 (0.05)	0.28 (0.02)	0.16 (0.05)	0.93 (0.02)	0.06 (0.04)	0.09 (0.04)	−0.13 (0.05)	.020*	−0.12 (0.05)	.010*
PGI-Education × Reunification | East Germany	0.33 (0.08)	0.28 (0.05)	0.36 (0.1)	0.87 (0.03)	−0.05 (0.07)	0.19 (0.07)	−0.33 (0.09)	< .001***	−0.17 (0.06)	.010*
PGI-Education × Region | Post Reunification	0.33 (0.08)	0.36 (0.06)	0.28 (0.1)	1.07 (0.04)	−0.25 (0.07)	0.08 (0.07)	−0.39 (0.11)	< .001***	−0.28 (0.12)	.020*
Birth-year analyses										
PGI-Education × Birth Year	0.20 (0.03)	0.34 (0.02)	0.10 (0.03)	0.95 (0.02)	0.04 (0.02)	0.08 (0.02)	−0.08 (0.03)	.010*	−0.08 (0.03)	< .001***
PGI-Education × Birth Year | East Germany	0.10 (0.06)	0.41 (0.04)	0.20 (0.06)	0.85 (0.03)	−0.03 (0.04)	0.21 (0.04)	−0.18 (0.05)	< .001***	−0.08 (0.04)	.020*

Note: Parameter estimates and standard errors in parentheses obtained from an environmental heteroscedasticity model are τ_1_, the main effect of the measured environment; π_0_, the main effect of PGI-Education, π_1_, the gene–environment interaction effect; λ_0_, the main effect of error term; and λ_E_, the interaction between the environment and error term, indexing environmental heteroscedasticity. The parameter estimate obtained from a genetic heteroscedasticity model is λ_G_, the interaction between PGI-Education and the error term indexing genetic heteroscedasticity. ξ_E_ and ξ_G_ are test statistics derived from the corresponding models (see Supplemental Methods in the Supplemental Material). Significant χ² test of ξ implicates that the gene–environment interaction is not driven by heteroscedasticity in the environment or PGI-Education. α = .05.

*** *p* < .001. *p < .05.

Second, we examined whether genetic associations with educational attainment significantly differed between East and West Germany in the full sample. They did not (*p* = .384; see Model 2 results in Table 3).

Third, we examined whether differences in genetic associations with educational attainment before versus after reunification differed between East and West Germany. The increase in education-genetic associations with educational attainment after reunification significantly differed by region (see Model 3 results in Table 3). Post hoc analyses that split the sample by region suggest that this interaction was driven by a postreunification amplification of the association between PGI-Education and educational attainment in East Germany (Reunification × PGI-Education: β = 0.38, 95% confidence interval, or CI = [0.18, 0.57], *p* < .001), which did not occur in West Germany (Reunification × PGI-Education: β = 0.10, 95% CI = [−0.03, 0.22], *p* = .14). See Figure S2 in the Supplemental Material for scatterplots.

To probe whether these results could be influenced by migration patterns related to PGI-Education, we regressed PGI-Education on region, reunification, and an interaction term Region × Reunification. We did not find a statistically significant main effect of region (*p* = .931) or a significant interaction effect between region and reunification (*p* = .147), indicating that PGI-Education did not significantly differ between East and West German samples pre- or postreunification (see the Supplemental Results).

**Fig. 1.**
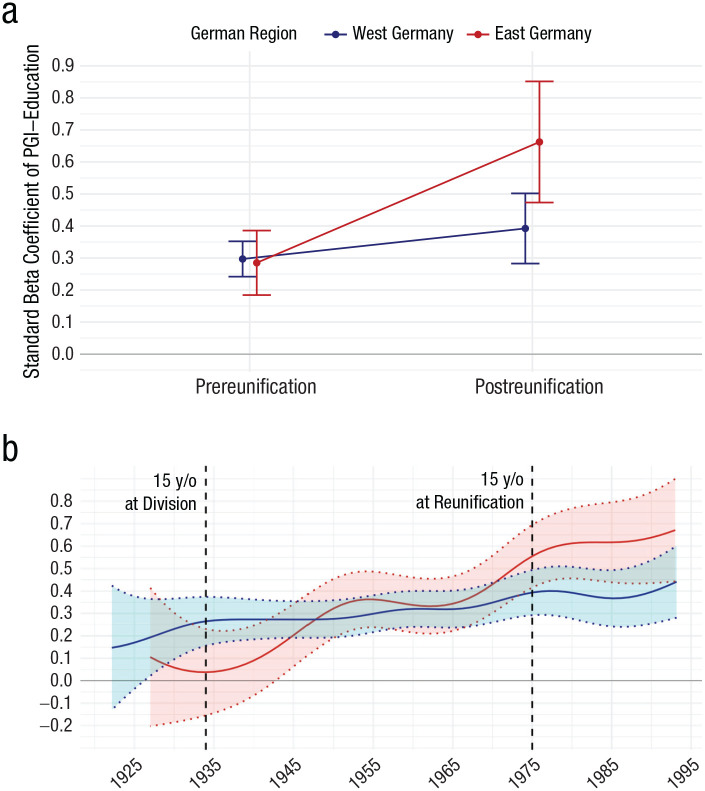
Effect size estimates of the association between PGI-Education and educational attainment in the German region around the time of reunification. Shown in (a) are the standardized beta coefficients and 95% confidence intervals of the association between PGI-Education (i.e., EA4) and years of education before and after German reunification (prereunification: *n*_west_ = 1,141, *n*_east_ = 353; postreunification: *n*_west_ = 329, *n*_east_ = 107). Error bars represent 95% confidence intervals (CIs). In (b) are shown the standardized beta coefficients and 95% CIs of the association between PGI-Education and educational attainment across birth years using weighted regressions. Coefficients derived from the West German sample are indicated in blue (*bw*_west_ = 4.96 years), whereas the East German sample is indicated in red (*bw*_east_ = 5.46 years). Individuals born before 1934 (i.e., left of the left vertical dashed line), were excluded from analyses because they turned 15 years old (y/o) before the formation of the German Democratic Republic in 1949. The right vertical dashed line divides cohorts that were older and younger than 15 years at the time of the German reunification in 1990 (*n*_west_ = 1,470, *n*_east_ = 460); *bw* = bandwidth.

Figure 1a plots the effect sizes of the association between PGI-Education and Years of Education by region and reunification. Although effect-size estimates were similar between East and West Germany before reunification (β_pre_ = 0.30, 95% CI = [0.24, 0.35], to β_post_ = 0.39, 95% CI = [0.28, 0.50]; *R*^2^_adjusted(pre)_ = .09, 95% CI = [.06, .12], to *R*^2^_adjusted(post)_ = .11, 95% CI = [.05, .18]), there was a substantial postreunification amplification of the predictive power of PGI-Education in East Germany (β_pre_ = 0.28, 95% CI = [0.18, 0.38], to β_post_ = 0.66, 95% CI = [.47, .85], *R*^2^_adjusted(pre)_ = .09, 95% CI = [.04, .18], to *R*^2^_adjusted(post)_ = .35, 95% CI = [.20, .47]; see Supplemental Results for line plot). We caution that small sample sizes, such as our postreunification subsample, tend to overestimate effect-size estimates (Hedges & Olkin, 1985). Given that raw *R*^2^ estimates are more positively biased with smaller samples, we report *R*^2^_adjusted_ to account for differences in sample size (Raju et al., 1997). Nevertheless, the postreunification effect size in East Germany should be interpreted cautiously and in appreciation of the uncertainty around the point estimate, as indicated by the confidence intervals.

### Birth-year analyses

Next, we examined whether the above gene–environment interaction replicated using a birth-year variable rather than German reunification. Consistent with the above results, the association between PGI-Education and educational attainment was amplified in younger Germans (see Model 1b results in Table 5). This gene–environment interaction remained significant when we applied a heteroscedasticity model (Table 4).

**Table 5.** Model Parameter Estimates Including Models With PGI-Education, Birth Year, and Region

Term	β	*SE*	*p*	95% CI
Model 1b: PGI-Education × Birth Year				
PGI-Education	0.34	0.03	< .001***	[0.29, 0.40]
Birth year	0.35	0.04	< .001***	[0.27, 0.43]
PGI-Education × Birth Year	0.11	0.03	< .001***	[0.05, 0.17]
*N* = 2025				
Model 3b: PGI-Education × Birth Year × East vs. West Germany				
PGI-Education	0.34	0.04	< .001***	[0.28, 0.42]
Birth year	0.36	0.04	< .001***	[0.27, 0.45]
Region (East Germany)	0.15	0.07	.039*	[0.01, 0.30]
PGI-Education × Birth Year	0.09	0.04	.038*	[0.00, 0.18]
PGI-Education × Region (East Germany)	0.06	0.07	.420	[−0.08, 0.20]
Birth Year × Region (East Germany)	0.02	0.10	.858	[−0.17, 0.21]
PGI-Education × Birth Year × Region (East Germany)	0.18	0.07	.011*	[0.04, 0.32]

Note: *N* = 1,930; *n*_east_ = 460, *n*_west_ = 1,470.

*** *p* < .001. *p < .05.

Second, we found that this increase in polygenic association with years of education in younger Germans differed by region (see Model 3b results in Table 5). Post hoc analyses that split the sample by region suggest that this interaction was driven by an amplification of genetic association in younger East Germans (Birth Year × PGI-Education: β = 0.26, 95% CI = [0.15, 0.37], *p* < .001), not younger West Germans (Birth Year × PGI-Education: β = 0.08, 95% CI = [0.01, 0.15], *p* = .03). Figure 1b plots the results of this analysis by regressing years of education on PGI-Education across different birth years on the basis of a nonparametric method. The bandwidth (*bw*) of the kernel-weighting function was set to 4.96 years for West Germans and 5.46 years for East Germans.

Models including both German reunification and birth-year interactions in the same model indicated that their effects are too collinear to distinguish (see the Supplemental Results).

To probe whether these results could be influenced by migration patterns related to PGI-Education, we regressed PGI-Education on region, birth year, and an interaction term Region × Birth Year. We did not find a statistically significant main effect of region (*p* = .372) or a significant interaction effect between region and birth year (*p* = .159), indicating that PGI-Education was not significantly different between East and West Germany in older or younger Germans (see the Supplemental Results).

### Negative control analysis

**Fig. 2.**
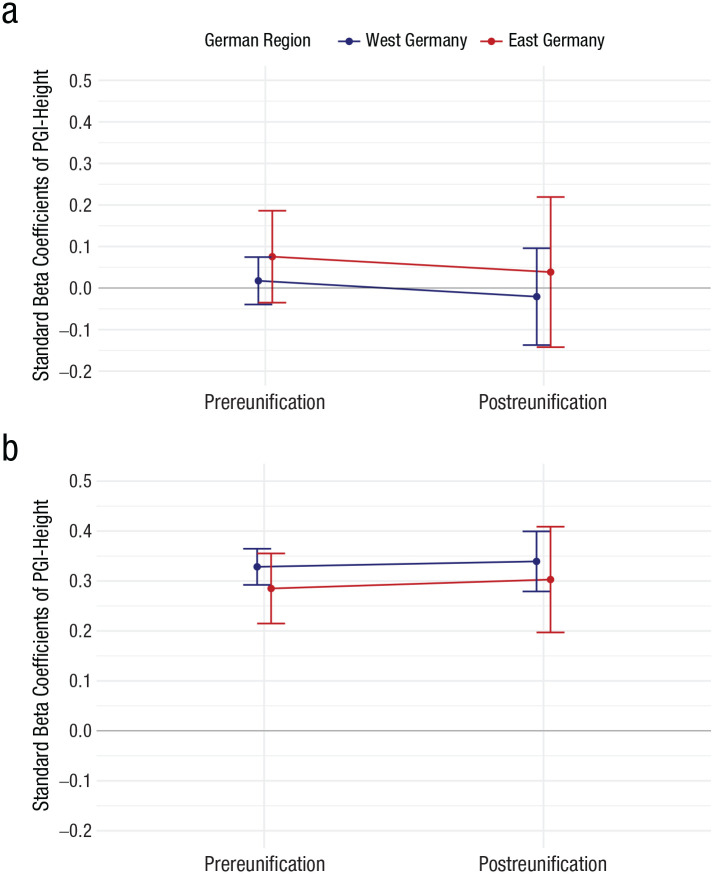
Effect-size estimates of the association between PGI-Height, educational attainment, and height by German region around the time of reunification. In (a) are plotted the standardized beta coefficients and 95% confidence intervals (CIs) of the association between PGI-Height and years of education before and after German reunification in East and West Germany. In (b) are plotted the standardized beta coefficients and 95% CIs of the association between PGI-Height and height before and after German reunification in East and West Germany.

To assess whether the findings were due to a more general pattern of varying genetic associations over time, we conducted negative control analyses using PGI-Height. Figure 2 shows that there was no significant main effect of PGI-Height on years of education (*p* = .725), and no significant interaction effects of PGI-Height with reunification (*p* = .882), region (*p* = .599), or three-way interaction effect with region and reunification *(p* = .993; see the Supplemental Results in the Supplemental Material). Moreover, PGI-height was significantly associated with self-reported height, β = 0.30, 95% CI = [0.26, 0.35], *p* < .001, but this association did not differ between before and after German reunification (*p* = .499) or between East and West Germany (*p* = .716). Additionally, there was no significant three-way interaction of PGI-Height with region and reunification (*p* = .924; see Table S6 in the Supplemental Results).

## Discussion

We explored geographical and historical differences in the association between measured DNA variants and educational attainment in East and West Germany around the time of reunification in 1,930 individuals aged 25 to 85 years from the SOEP-G cohort. We found that the magnitude of the association between PGI-Education and educational attainment did not differ between East and West Germany before reunification and was amplified in postreunification East Germany, which experienced a profound transition from a socialist state to a pluralist and meritocratic ideology oriented toward free-market productivity (Littler, 2017; Rohde, 2023; Solga, 2005). Notably, prior to reunification, associations were of a very similar magnitude in East and West Germany, even though the PGI-education is based on weights obtained from analyses in free-market Western democracies. The postreunification gene–environment interaction remained significant when we applied a heteroscedasticity model probing potential effects of dispersion in phenotypic variance (Domingue et al., 2022). Moreover, negative control analyses of a polygenic index of height with educational attainment and self-reported height indicated that this gene–environment interaction is unlikely to reflect a general pattern of varying genetic associations over time. In addition, our time-concurrent regional contrast between East and West Germany substantially reduces the likelihood of cohort-related confounds that could affect genetic associations.

Collectively, these findings are more consistent with the interpretation that there was an amplification of education-genetic associations in East Germany after German reunification rather than a socialist-era suppression of genetic effects relative to West Germany. This aligns with previous reports that the association between PGI-Education and educational attainment was amplified in Estonia and Hungary after the collapse of the Soviet Union in Europe (Rimfeld et al., 2018; Ujma et al., 2022). In comparison to these previous studies—Estonia (EA2): *R*^2^_post-soviet_ ~ 3.8%; Hungary (EA3): *R*^2^_post-soviet_ ~ 4.0%—we observed a notably large effect-size estimate of polygenic associations in postreunification East Germany of *R*^2^_adjusted_ = 34.7%. We highlight the wide confidence intervals around this parameter estimate (95% CI = [20.0, 46.6]) and caution that effect-size estimates are more positively biased in smaller samples (Hedges & Olkin, 1985). One difference between the present study and previous studies is that the most recent version of PGI-Education (EA4) used here is typically more strongly associated with educational attainment than earlier iterations of the PGI-Education probed in these earlier studies because they are constructed on the basis of results from a more statistically powerful GWAS (EA4: *N* > 3 million, *R*^2^ = 15.8%, 95% CI = [14, 17]; Okbay et al., 2022; compared to EA3: *N* = 1.1 million, *R*^2^ = 12.6%, 95% CI = [10.9, 14.5]; Lee et al., 2018). Moreover, a substantial portion of “hidden heritability” for education (i.e., the gap between estimated heritability and polygenic index effect sizes) has been shown to be due to heterogeneity across populations and time periods (Tropf et al., 2017). Consequently, we might anticipate larger PGI effect sizes when samples are highly homogenous, as in our postreunification East German sample.

One potential explanation for the large interaction effect is that it is an artifact of the mass migration between East and West Germany following the opening of the borders. In other countries, such as the United Kingdom and Estonia, PGI-Education has been associated with an individual’s propensity to migrate to more affluent regions (Abdellaoui et al., 2019; Kuznetsov et al., 2023). We probed this hypothesis by examining potential differences in PGI-Education between East and West Germany and found no evidence for differences either before or after reunification, meaning that individuals with higher PGI-Education were not more likely to be born in or to migrate to West Germany. Nevertheless, examining education-genetic differences in relation to intra-German migration following reunification remains an interesting avenue for future research in larger German genetic data sets that can also provide more robust effect-size estimates.

We speculate that two main factors—ideological selection and intergenerational educational inequality—may have contributed to the East–West differences in education-genetic associations around reunification. First, the restrictive state-socialist ideology that discriminated against students who did not align with the socialist agenda was rapidly and completely replaced by Western pluralist and meritocratic principles (Rimfeld et al., 2018; von Below, 1997). Second, intergenerational educational inequality was lower in East relative to West Germany before and shortly after reunification, before it gradually approached West German levels (see Box S1 in the Supplemental Material; Engzell & Tropf, 2019; Klein et al., 2019). After the sudden fall of the socialist elites and initial absence of a wealthy class, East Germany’s new education system, based on merit, did not favor any specific social group, unlike in West Germany. As a result, the postreunification generation of East Germans found themselves uniquely liberated from the constraints of ideological selection and parental social status (Klein et al., 2019). Generally speaking, genetic influences on educational behaviors have been hypothesized to increase during times of transition characterized by more social, educational, and economic choices (Briley et al., 2015; Engzell & Tropf, 2019; Raffington et al., 2020; Tucker-Drob et al., 2013).

We acknowledge that polygenic indices of educational attainment do not capture *all* education–genetic effects and reflect a mixture of direct genetic influence (e.g., one’s disposition to academic persistence, primary marking effects of social origin), indirect genetic influence (e.g., parental-nurture effects on child education, secondary marking effects of social origin), and also socially stratified environmental differences between families (e.g., dynastic social processes, tertiary marking effects of social origin; Aikins et al., 2024; Helbig & Morar, 2018; Malanchini et al., 2024; Nivard et al., 2024; Wertz et al., 2023). It is possible that genetic propensities for traits such as political conformity were more relevant to educational attainment in prereunification East Germany and that these factors are not captured by this polygenic index. Yet our finding that genetic associations were similar between East and West Germany before reunification is more consistent with the interpretation that genetic influences became more important during this social transition than the interpretation that different genes mattered in East versus West Germany. Future studies could corroborate this interpretation by probing whether educational performance and aspirations were more predictive of educational attainment in East Germany compared with West Germany shortly after reunification.

A meritocratic-oriented educational system may have both desirable and unwanted effects on social justice and cohesion. It is generally considered desirable for a society to have individuals who excel in roles such as caregivers, public servants, and pilots. Thus, matching genetically influenced skills and preferences to corresponding educational training and vocations is beneficial for society at large and may reflect a more equal society that does not restrict access to education on the basis of class, gender, or similar (Raffington et al., 2020). Yet meritocratic selection that leads to substantially higher monetary and health rewards on the basis of genetically influenced performance and aspirations could have unintended consequences that amplify intergenerational social inequality and threaten social cohesion (Bloodworth, 2016; Harden, 2021; Knigge et al., 2022; Rimfeld et al., 2018). Our study provides evidence that individual genetic predictors, previously developed in Western democratic countries, were associated with educational attainment even in the state-socialist educational system of East Germany. This tempers expectations that genetically influenced differences in educational performance will be readily eliminated by incremental changes in educational policy within free-market democracies. Tackling the downstream individual and intergenerational inequities in income, health, and life opportunities that arise from differences in educational attainment thus remains of utmost importance to foster social justice and coherence in meritocratic-oriented societies.

## Supplemental Material

sj-docx-1-pss-10.1177_09567976251350965 – Supplemental material for Polygenic Associations With Educational Attainment in East Versus West Germany: Differences Emerge After Reunification Supplemental material, sj-docx-1-pss-10.1177_09567976251350965 for Polygenic Associations With Educational Attainment in East Versus West Germany: Differences Emerge After Reunification by Deniz Fraemke, Yayouk E. Willems, Aysu Okbay, Ulman Lindenberger, Sabine Zinn, Gert Wagner, David Richter, Kathryn P. Harden, Elliot M. Tucker-Drob, Ralph Hertwig, Philipp Koellinger and Laurel Raffington in Psychological Science

## Transparency

*Action Editor:* D. A. Briley

*Editor:* Simine Vazire

*Author Contributions*

**Deniz Fraemke:** Conceptualization; Data curation; Formal analysis; Investigation; Methodology; Project administration; Software; Validation; Visualization; Writing – original draft; Writing – review & editing.

**Yayouk E. Willems:** Methodology; Software; Validation; Writing – review & editing.

**Aysu Okbay:** Data curation; Resources.

**Ulman Lindenberger:** Methodology; Supervision; Supervision; Writing – review & editing; Writing – review & editing.

**Sabine Zinn:** Resources; Writing – review & editing.

**Gert Wagner:** Data curation; Funding acquisition; Resources.

**David Richter:** Data curation; Resources; Supervision; Writing – review & editing.

**Kathryn P. Harden:** Formal analysis; Methodology; Writing – review & editing.

**Elliot M. Tucker-Drob:** Formal analysis; Methodology; Writing – review & editing.

**Ralph Hertwig:** Data curation; Resources; Writing – review & editing.

**Philipp Koellinger:** Data curation; Resources; Writing – review & editing.

**Laurel Raffington:** Conceptualization; Data curation; Formal analysis; Funding acquisition; Investigation; Methodology; Project administration; Resources; Software; Supervision; Validation; Visualization; Writing – original draft; Writing – review & editing.

**Artificial Intelligence:** No artificial-intelligence-assisted technologies were used in this research or the creation of this article.

**Ethics:** The data collection for this study has received ethical approval by the Vrije Universiteit Amsterdam, School of Business and Economics (Application No. 20181018.1.pkr730) and by the Max Planck Society (Application No. 2019_16).

**Open Practices:** Preregistration: No aspect of this study was preregistered. Materials: The SOEP-IS is a longitudinal panel study initiated in 1998 that collects data annually. All questionnaires and summary statistics of each item used in this study are provided in the data documentation of the SOEP-IS (https://paneldata.org/soep-is/). Data: All data used in this study are fully accessible to eligible researchers upon application to the management of the DIW Berlin. For more details see https://osf.io/baehf. Analysis Scripts: All analysis scripts are publicly available (https://osf.io/8skmg/). Computational reproducibility: The reproducibility of this manuscript was not verified by the journal’s STAR team because it was not possible to access the data within a reasonable time frame. The original analyses were run by D. Fraemke and then independently reproduced by Y. E. Willems.
