# Supplementary material for: Polygenic Associations With Educational Attainment in East Versus West Germany: Differences Emerge After Reunification
Source: Psychol Sci. 2025 Jul 9;36(7):559–73. doi: 10.1177/09567976251350965 (PMC13043040; doi:10.1177/09567976251350965)
Supplement: sj-docx-1-pss-10.1177_09567976251350965 – Supplemental material for Polygenic Associations With Educational Attainment in East Versus West Germany: Differences Emerge After Reunification [file sj-docx-1-pss-10.1177_09567976251350965.docx]

**Supplemental Information**

| **Box 1**. Differences Between East and West German Educational Systems  We speculate that two main factors - ideological selection and intergenerational educational inequality - may have contributed to East-West differences in education-genetic associations around reunification. Here we provide more information on these factors. First, educational systems differed significantly in the extent to which they prioritized the influence of ideological values on the education system. The East German school system was centrally directed by the state government and had the primary principle of educating students to ‘holistic socialist personalities’^1^. As a manifestation of this principle, ideological conformity influenced admission to upper secondary school and university. Students involved in state youth groups, such as the Young Pioneers and the Free German Youth (“*Freie Deutsche Jugend”*, FDJ) or those showing high academic performance in Marxist-Leninist civics education, were favored. Conversely, religious activities, familial connections to West Germany, non-conformist attitudes, or refusal to join the ruling Socialist Unity Party of Germany (“*Sozialistische Einheitspartei Deutschlands”, SED*) were grounds to deny admission (Fischer, 1992).  In contrast, ideological and social pluralism within constitutional boundaries were fundamental to the federalist education system in West Germany. This refers to both the pluralism of ideological, religious and political directions that were taught and supported, as well as the pluralism in the participation of groups involved in education (*e.g.,* employers' associations or trade unions; Anweiler, 1990). Additionally, the West German school system differs by the core principle of meritocracy (“*Leistungsprinzip”*), which is widespread among teachers and students (Rohde, 2023).  Second, intergenerational educational inequality differed substantially between East and West Germany. The East German state socialist regime was committed to an egalitarian socio-political program. From 1946 onwards, large-scale institutional reforms aimed at transforming the educational system to abolish discrimination based on gender and class differences were implemented (Anweiler, 1990; Fischer, 1992; von Below, 2017). As a result, intergenerational inequality was significantly lower in East Germany during the years of state socialism, and converged to West German levels several years after reunification (Klein et al., 2019). The introduction of a comprehensive school system, extending until the end of the 10th grade (~ age 16 years), was designed to equalize abilities among students. As a result, low-achieving students received significantly more academic support compared to their high-achieving peers (Anweiler, 1989).  In West Germany, the postulate of social equality of opportunity (“*Chancengleichheit”*) first gained attention in the 1960s, which was much later than in the East. The implementation of directive or restrictive measures with the objective of reducing social inequalities was generally rejected. Systematically ‘redistributing opportunities’ through administrative measures was considered contrary to constitutional norms (Anweiler, 1990). As a result, West Germany exhibits pronounced inequalities in education when compared to other countries (Schnepf, 2002). For instance, quasi-experimental evidence suggests that early school tracking in grade 4 (~ age 10 years) reproduces intergenerational educational inequality (Schneider, 2008; Strello et al., 2021). Early school tracking assigns students into lower, intermediate, or upper secondary school tracks based on primary school academic performance. Transitions between tracks are uncommon, and university admission is only possible for students with a degree from the higher track (Anweiler, 1990).  A promising approach to reducing educational inequality in West Germany emerged in the 1960s with the establishment of comprehensive schools, designed to ensure equitable access across social classes while allowing internal differentiation based on individual performance and abilities (e.g., through course selection systems). Additionally, mandatory preschool education from age five has demonstrated potential in mitigating educational disparities (Schlicht et al., 2010). |
| --- |
| ^1^1§ (1) of GDR’s Education Act (1965): “The aim of the **unified socialist educational system** is a high level of education for the entire population, the education and upbringing of comprehensively and **holistically developed socialist personalities**, who consciously shape societal life, alter nature, and lead a fulfilled, happy, and dignified life”. |

**Supplemental Methods**

**Genetic data**

Buccal swabs and Isohelix IS SK-1S Dri-Capsules were used to collect DNA data. DNA extraction and methylation profiling were conducted at the Erasmus Medical Center in the Netherlands by the Human Genomics Facility (HuGe-F). The use of the DNA samples received ethical approval by The Vrije Universiteit Amsterdam, School of Business and Economics (application number 20181018.1.pkr730) and the Max Planck Society (application number 2019_16). Genotyping was conducted using the Illumina Infinium Global Screening Array-24 v3.0 BeadChips. Genotypes were subject to quality control excluding participants with sex mismatch, with per-chromosome missingness of more than 50%, and with excess heterozygosity/homozygosity.

The Haplotype Reference Consortium reference panel (r.1.1) for imputation was used with imputation accuracy (R^2^) greater than 0.1. Approximately 66% of the imputed SNPs were rare with minor allele frequencies (MAF) smaller than 0.01 and ~24% SNPs were common. The average imputation accuracy in the data was 0.66, with higher imputation accuracy for common SNPs (MAF>0.05) with an average imputation accuracy of 0.92. To control for population stratification, the first 20 principal components (PCs) were computed for individuals with high genetic similarity to European reference groups, based on ~160,000 approximately independent SNPs with imputation accuracy ≥ 70% and MAF ≥ 0.01 (see Koellinger et al., 2023).

**Heteroscedasticity**

We applied the methods developed by Domingue et al. (2022), to directly model the dispersion of the outcome and test whether a GxE is specific to a measured predictor or represents a general pattern of variation in the outcome. We extend the general linear model in Equation 1 with a coefficient (*λ_E_* or *λ_G_*), estimating the interaction between the error term and the respective environmental or genetic predictor, which indexes heteroscedasticity. Equation 1 indicates an example environmental model.

|  | *Y_i_* =*𝜏*_0_ +*𝜏*_1_*E_i_* +*𝜋*_0_*G_i_* +*𝜋*_1_*G_i_ E_i_* +*𝜆*_0_*𝜖_i_* +*𝜆*_E_*E_i_* ⋅*𝜖_i_*. | (1) |
| --- | --- | --- |

Here, *𝜏*_0_ is the intercept, *𝜏_1_* represents the parameter of the main effect of the measured environment, *𝜋_0_* the main effect of the genetic predictor, *𝜋_1_* the GxE interaction effect and *𝜆_0_* the main effect of the error term. In a genetic heteroscedasticity model, the *𝜆*_E_ is replaced by a *𝜆*_G_ and would model the interaction between *G_i_* and the error term. Following Equation 2 and 3, we derive the test statistics *ξ*_E_ and *ξ*_G_ from the corresponding models.

|  | ${}_{E} \hat{\pi_{0}}\hat{{}_{E}}-\hat{\pi_{1}}\hat{{}_{0}}$ | (2) |
| --- | --- | --- |
|  | ${}_{G} \hat{\tau_{0}}\hat{{}_{G}}-\hat{\pi_{1}}\hat{{}_{0}}$ | (3) |

The authors show that if a *χ²*-test fails to reject *H*_0_ ∶ *ξ* = 0*,* it cannot be ruled out that the interaction is driven by dispersion in the outcome over the respective environmental or genetic predictor. When the test suggests rejection of *H*_0_∶ *ξ*= 0, alternative forms of GxE are implicated.

**Supplemental Results**

In a model that included both German reunification x PGI-Education x East/West and birth year x PGI-Education x East/West interactions, neither of the interactions was statistically significant (**Table S1**). This indicates that the effects of German reunification and birth year are too collinear to distinguish.

We conducted several sensitivity analyses to probe the robustness of our main results. We examined if genetic relatedness within families or genotyping quality introduced unwanted bias in the results. Excluding individuals who had either siblings or parents in the sample (n = 16) did not affect results (**Table S2**). Excluding individuals that had not passed a strict genotyping quality control (QC) pipeline (n = 172; Koellinger et al., 2023) did not substantially affect model parameters (**Table S3**). Moreover, the inclusion of BMI as a covariate did not substantially affect the results (**Table S4**).

Next, we present the full results of the negative control analyses using a polygenic index of height (Wood et al., 2014) with educational attainment and height, which tests for a pattern of generally elevated genetic influence. There were no associations of PGI-Height with educational attainment (**Table S5**). Moreover, associations of PGI-Height with self‑reported height did not differ between pre- and post-reunification, nor between East and West Germany (**Table S6**). Lastly, we conducted migration related analyses, where we probed differences in PGI-Education between East and West Germany after the reunification, discussed in the main text (**Table S7**).

| **Table S1.**  Full model results with both German reunification and birth year in one model. | | | | |
| --- | --- | --- | --- | --- |
| **Term** | **β** | **SE** | ***p*** | **95% CI** |
| PGI-Education x Birth Year x East vs. West Germany and  PGI-Education x Reunification x East vs. West Germany | | | | |
| PGI-Education | 0.38 | 0.05 | < .001*** | [0.28, 0.48] |
| Region (East Germany) | 0.04 | 0.12 | .727 | [-0.19, 0.27] |
| PGI-Education ×  Region (East Germany) | 0.03 | 0.10 | .746 | [-0.16, 0.22] |
| Reunification Terms |  |  |  |  |
| Reunification (Post) | -0.12 | 0.13 | .340 | [-0.38, 0.13] |
| Reunification (Post) ×  PGI-Education | -0.12 | 0.12 | .325 | [-0.36, 0.12] |
| Reunification (Post) ×  Region (East Germany) | 0.34 | 0.27 | .201 | [-0.18, 0.86] |
| Reunification (Post) ×  PGI-Education ×  Region (East Germany) | 0.10 | 0.20 | .624 | [-0.30, 0.49] |
| Birth Year Terms |  |  |  |  |
| Birth Year | 0.41 | 0.07 | < .001*** | [0.27, 0.56] |
| Birth Year ×  PGI-Education | 0.14 | 0.07 | .039* | [0.01, 0.28] |
| Birth Year ×  Region (East Germany) | -0.13 | 0.15 | .393 | [-0.43, 0.17] |
| Birth Year ×  PGI-Education ×  Region (East Germany) | 0.15 | 0.12 | .201 | [-0.08, 0.37] |
|  | | | | |

| **Table S2**  Full model results in unrelated individuals. | | | | |
| --- | --- | --- | --- | --- |
| **Term** | **β** | **SE** | ***p*** | **95% CI** |
| PGI-Education x Reunification x East vs. West Germany | | | | |
| PGI-Education | 0.29 | 0.04 | < .001*** | [0.22, 0.36] |
| Reunification (Post) | 0.48 | 0.08 | < .001*** | [0.32, 0.64] |
| Region (East Germany) | 0.11 | 0.08 | .168 | [-0.04, 0.26] |
| PGI-Education × Reunification (Post) | 0.11 | 0.08 | .159 | [-0.04, 0.27] |
| PGI-Education ×  Region (East Germany) | -0.04 | 0.07 | .553 | [-0.19, 0.10] |
| Reunification (Post) × Region (East Germany) | 0.12 | 0.17 | .471 | [-0.21, 0.46] |
| PGI-Education × Reunification (Post) × Region (East Germany) | 0.27 | 0.13 | .030* | [0.03, 0.52] |
| *Note.* N = 1914. | | | | |

| **Table S3**  Full model results in a subsample passing strict DNA quality control. | | | | |
| --- | --- | --- | --- | --- |
| **Term** | **β** | **SE** | ***p*** | **95% CI** |
| PGI-Education x Reunification x East vs. West Germany | | | | |
| PGI-Education | 0.30 | 0.04 | < .001*** | [0.22, 0.38] |
| Reunification (Post) | 0.48 | 0.08 | < .001*** | [0.32, 0.64] |
| Region (East Germany) | 0.12 | 0.08 | .127 | [-0.04, 0.28] |
| PGI-Education × Reunification (Post) | 0.12 | 0.08 | .159 | [-0.05, 0.28] |
| PGI-Education ×  Region (East Germany) | -0.06 | 0.08 | .450 | [-0.21, 0.09] |
| Reunification (Post) × Region (East Germany) | 0.12 | 0.18 | .484 | [-0.22, 0.47] |
| PGI-Education × Reunification (Post) × Region (East Germany) | 0.27 | 0.13 | .034* | [0.02, 0.52] |
| *Note.* N = 1770. Model parameter after excluding individuals with samples that did not pass strict quality control of genetic data. We controlled for the two covariates gender, BMI and PGI-Education x covariate, reunification x covariate and region x covariate interactions with both covariates (cf. Keller, 2014). | | | | |

| **Table S4**  Full model results with covariate control for body mass index. | | | | |
| --- | --- | --- | --- | --- |
| **Term** | **β** | **SE** | ***p*** | **95% CI** |
| **Model 3:** PGI-Education x Reunification x East vs. West Germany | | | | |
| PGI-Education | 0.26 | 0.04 | < .001*** | [0.19, 0.34] |
| Reunification (Post) | 0.45 | 0.08 | < .001*** | [0.29, 0.62] |
| Region (East Germany) | 0.12 | 0.08 | .107 | [-0.03, 0.28] |
| PGI-Education × Reunification (Post) | 0.08 | 0.08 | .343 | [-0.09, 0.24] |
| PGI-Education ×  Region (East Germany) | 0.01 | 0.07 | .931 | [-0.14, 0.15] |
| Reunification (Post) × Region (East Germany) | 0.12 | 0.17 | .493 | [-0.22, 0.46] |
| PGI-Education × Reunification (Post) × Region (East Germany) | 0.27 | 0.13 | .031* | [0.02, 0.52] |
| *Note.* We controlled for the covariate gender and BMI, as well as all PGI-Education x covariate, Reunification x covariate and Region x covariate, and all PGI-Education x Reunification x covariate, PGI-Education x Region x covariate and Reunification x Region x covariate interactions (cf. Keller, 2014). | | | | |

| **Table S5**  *Full model results of PGI-Height on Years of Education* | | | | | |
| --- | --- | --- | --- | --- | --- |
| **Term** | **β** | **SE** | **p** | | **95% CI** |
| PGI-Height x Reunification x East vs. West Germany | | | | | |
| PGI-Height | 0.01 | 0.04 | .725 | [-0.06, 0.09] | |
| Reunification (Post) | 0.10 | 0.08 | .210 | [-0.06, 0.26] | |
| Region (East Germany) | 0.38 | 0.09 | < .001*** | [0.21, 0.55] | |
| PGI-Height ×  Reunification (Post) | 0.01 | 0.07 | .882 | [-0.14, 0.16] | |
| PGI-Height ×  Region (East Germany) | -0.04 | 0.08 | .599 | [-0.21, 0.12] | |
| Reunification × Region | -0.04 | 0.18 | .828 | [-0.39, 0.31] | |
| PGI-Height ×  Reunification (Post) ×  Region (East Germany) | 0.00 | 0.13 | .993 | [-0.25, 0.25] | |
| *Note.* We controlled for the covariate gender, PGI-Height x gender, reunification x gender and Region x gender (cf. Keller, 2014). | | | | | |

| **Table S6**  *Full model results of PGI-Height on self-reported Height* | | | | | |
| --- | --- | --- | --- | --- | --- |
| **Term** | **β** | **SE** | **p** | | **95% CI** |
| PGI-Height x Reunification x East vs. West Germany | | | | | |
| PGI-Height | 0.30 | 0.02 | < .001*** | [0.26, 0.35] | |
| Reunification (Post) | -0.24 | 0.05 | < .001*** | [-0.34, -0.14] | |
| Region (East Germany) | 0.25 | 0.05 | < .001*** | [0.15, 0.34] | |
| PGI-Height ×  Reunification (Post) | 0.03 | 0.05 | .499 | [-0.06, 0.12] | |
| PGI-Height ×  Region (East Germany) | -0.02 | 0.04 | .716 | [-0.10, 0.07] | |
| Reunification × Region | 0.26 | 0.10 | .011* | [0.06, 0.47] | |
| PGI-Height ×  Reunification (Post) ×  Region (East Germany) | 0.01 | 0.07 | .924 | [-0.14, 0.15] | |
| *Note.* We controlled for the covariate gender, PGI-Height x gender, reunification x gender and Region x gender (cf. Keller, 2014). | | | | | |

| **Table S7**  *Full model results of PGI-Education on East vs. West & Pre vs. Post-Reunification* | | | | |
| --- | --- | --- | --- | --- |
| **Term** | **β** | **SE** | ***p*** | **95% CI** |
| **Migration Model 1:** Region x Reunification | | | | |
| Reunification (Post) | -0.23 | 0.06 | < .001*** | [-0.35, -0.10] |
| Region (East Germany) | -0.01 | 0.07 | .931 | [-0.15, 0.14] |
| PGI-Education ×  Region (East Germany) | -0.15 | 0.11 | .147 | [-0.36, 0.05] |
| **Migration Model 2**: Region x Birth Year | | | | |
| Birth Year | -0.12 | 0.03 | < .001*** | [-0.18, -0.06] |
| Region (East Germany) | -0.06 | 0.07 | .372 | [-0.19, 0.07] |
| Birth Year ×  Region (East Germany) | -0.07 | 0.05 | .159 | [-0.17, 0.03] |
| *Note.* N = 2313. We controlled for the covariate gender, Reunification x gender and Region x gender (cf. Keller, 2014). | | | | |

**
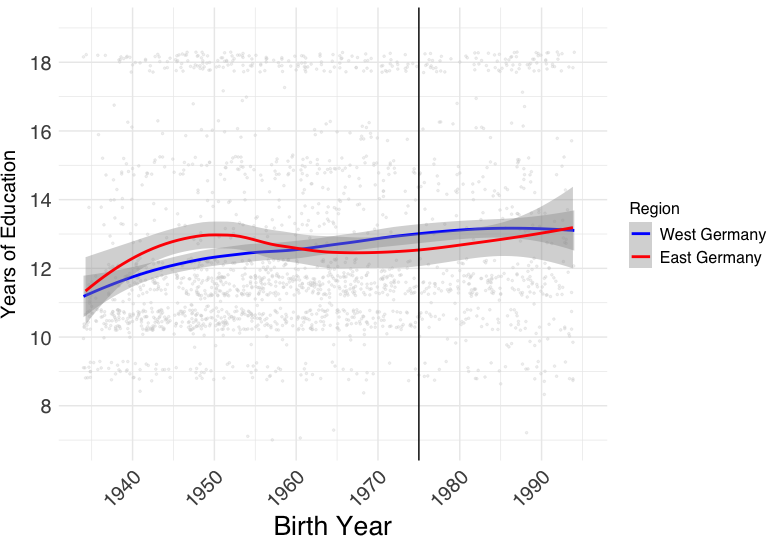
**

**Figure S1.** **Years of Education by birth year in East and West Germany.** The vertical line separates individuals who turned 15 years before and after German reunification in 1990. Clustering random noise is added to years of education in this figure for visualization purposes.

East Germany

West Germany

| Before German reunification  After German reunification |
| --- |
| 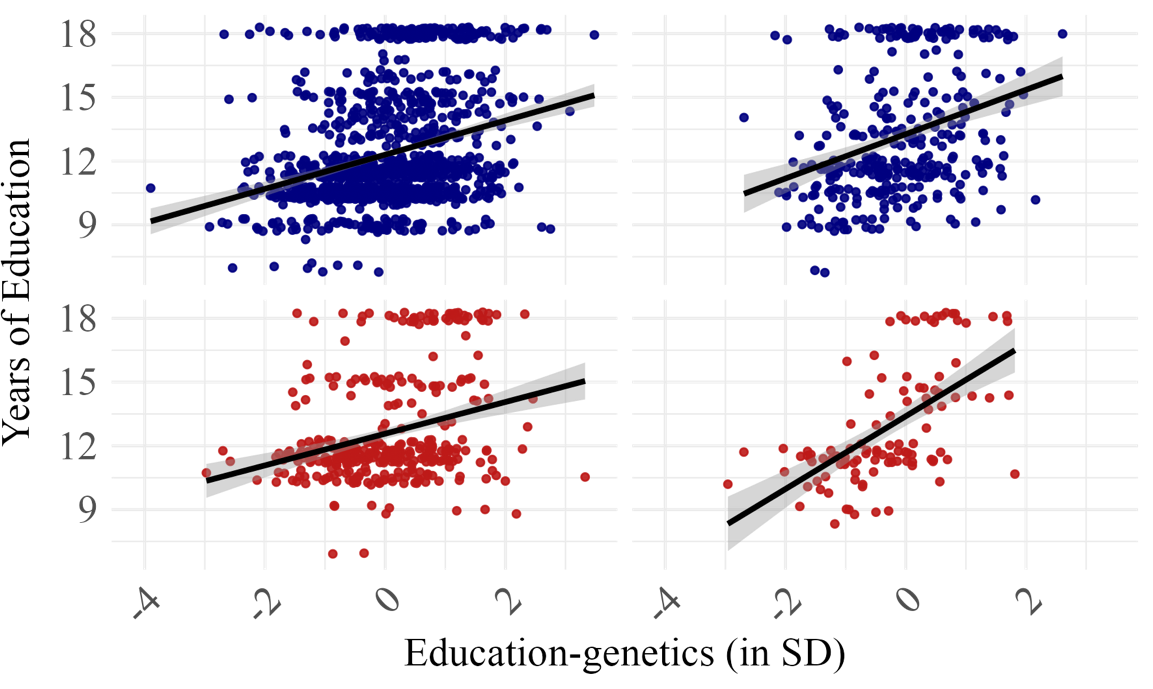 |
| **Figure S2. Scatterplot of the association between PGI-Education and educational attainment by region before and after German reunification in East and West Germany.** |

Educational attainment incremental R² (%)

| **Figure S3**  *Effect size estimates of the association between PGI-Education and educational attainment by region and time period.* | | |
| --- | --- | --- |
| 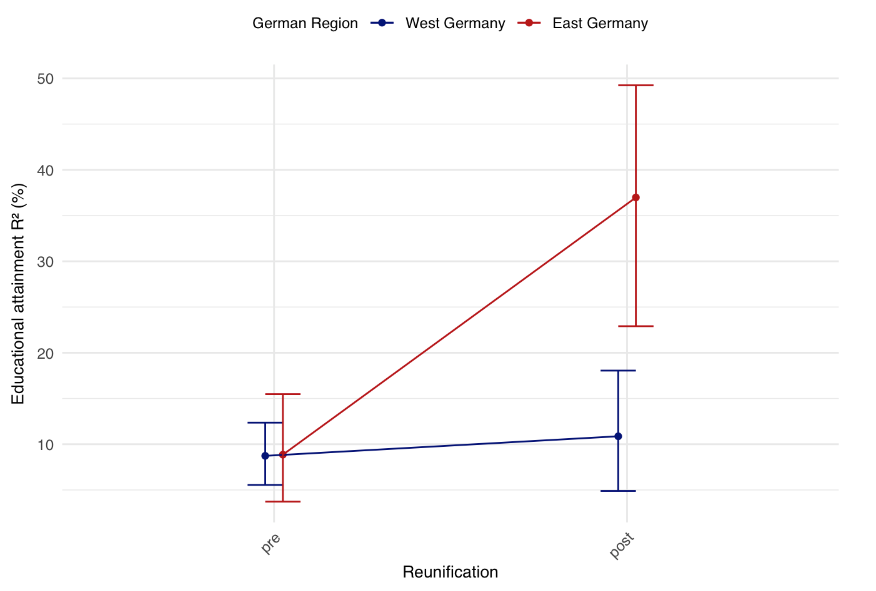 | |  |
| *Note*. Adjusted R^2^ of PGI-Education (i.e., EA4) and educational attainment before and after German reunification in East and West Germany. Error bars indicate 95% bootstrapped confidence intervals with 2,000 replications. (Pre-Reunification: *N*_west_ = 1141, *N*_east_ = 353; Post-Reunification: *N*_west_ = 329; *N*_east_ = 107). We caution that small sample sizes, such as our post-reunification subsample, tend to overestimate effect size estimates (Hedges & Olkin, 1985). |  |  |

**References**

Anweiler, O. (1989). *Schulpolitik und Schulsystem in der DDR*. VS Verlag für Sozialwissenschaften. https://doi.org/10.1007/978-3-322-95525-8

Anweiler, O. (1990). *Vergleich von Bildung und Erziehung in der Bundesrepublik Deutschland und in der Deutschen Demokratischen Republik*. Verlag Wissenschaft und Politik.

Domingue, B. W., Kanopka, K., Mallard, T. T., Trejo, S., & Tucker-Drob, E. M. (2022). Modeling Interaction and Dispersion Effects in the Analysis of Gene-by-Environment Interaction. *Behavior Genetics*, *52*(1), 56–64. https://doi.org/10.1007/s10519-021-10090-8

Fischer, A. (1992). *Das Bildungssystem der DDR: Entwicklung, Umbruch und Neugestaltung seit 1989*. Wissenschaftliche Buchgesellschaft.

Hedges, L. V., & Olkin, I. (1985). *Statistical Methods for Meta-Analysis*. Academic Press.

Klein, M., Barg, K., & Kühhirt, M. (2019). Inequality of Educational Opportunity in East and West Germany: Convergence or Continued Differences? *Sociological Science*, *6*, 1–26. https://doi.org/10.15195/v6.a1

Koellinger, P. D., Okbay, A., Kweon, H., Schweinert, A., Linnér, R. K., Goebel, J., Richter, D., Reiber, L., Zweck, B. M., Belsky, D. W., Biroli, P., Mata, R., Tucker-Drob, E. M., Harden, K. P., Wagner, G., & Hertwig, R. (2023). Cohort profile: Genetic data in the German Socio-Economic Panel Innovation Sample (SOEP-G). *PLOS ONE*, *18*(11), e0294896. https://doi.org/10.1371/journal.pone.0294896

Rohde, N. (2023). ‘To assign people their place in society’: School grades and the quantification of merit. *Economy and Society*, *52*(3), 506–530. https://doi.org/10.1080/03085147.2023.2225346

Schlicht, R., Stadelmann-Steffen, I., & Freitag, M. (2010). Educational Inequality in the EU: The Effectiveness of the National Education Policy. *European Union Politics*, *11*(1), 29–59. https://doi.org/10.1177/1465116509346387

Schneider, T. (2008). Social Inequality in Educational Participation in the German School System in a Longitudinal Perspective: Pathways into and out of the most Prestigious School Track. *European Sociological Review*, *24*(4), 511–526. https://doi.org/10.1093/esr/jcn017

Schnepf, S. V. (2002). *A Sorting Hat that Fails? The Transition from Primary to Secondary School in Germany*. *92*.

Strello, A., Strietholt, R., Steinmann, I., & Siepmann, C. (2021). Early tracking and different types of inequalities in achievement: Difference-in-differences evidence from 20 years of large-scale assessments. *Educational Assessment, Evaluation and Accountability*, *33*(1), 139–167. https://doi.org/10.1007/s11092-020-09346-4

von Below, S. (2017). Bildungssysteme im historischen und internationalen Vergleich. In R. Becker (Ed.), *Lehrbuch der Bildungssoziologie* (pp. 151–177). Springer Fachmedien Wiesbaden. https://doi.org/10.1007/978-3-658-15272-7_5

Wood, A. R., Esko, T., Yang, J., Vedantam, S., Pers, T. H., Gustafsson, S., Chu, A. Y., Estrada, K., Luan, J., Kutalik, Z., Amin, N., Buchkovich, M. L., Croteau-Chonka, D. C., Day, F. R., Duan, Y., Fall, T., Fehrmann, R., Ferreira, T., Jackson, A. U., … Frayling, T. M. (2014). Defining the role of common variation in the genomic and biological architecture of adult human height. *Nature Genetics*, *46*(11), Article 11. https://doi.org/10.1038/ng.3097
